# Future-proofing the primary care workforce: A qualitative study of home visits by emergency care practitioners in the UK

**DOI:** 10.1080/13814788.2021.1909565

**Published:** 2021-05-12

**Authors:** Robert Oliver Barker, Rachel Stocker, Siân Russell, Barbara Hanratty

**Affiliations:** Population Health Sciences Institute, Newcastle University, Newcastle-upon-Tyne, United Kingdom

**Keywords:** General practice/family medicine, quality of care, integrated care, qualitative designs and methods

## Abstract

**Background:**

Broadening the skill-mix in general practice is advocated to build resilience into the primary care workforce. However, there is little understanding of how extended-scope practitioners from different disciplines, such as paramedicine and nursing, embed into roles traditionally ascribed to general practitioners (GPs).

**Objectives:**

This study sought to explore patients' and professionals' experiences of a primary care home visiting service delivered by emergency care practitioners (ECPs), in place of GPs; to determine positive impacts/unintended consequences and establish whether interdisciplinary working was achieved.

**Methods:**

Three practices in England piloted an ECP (extended-scope practitioners with a paramedic or nursing background) home visiting service (November 2018–March 2019). Following the pilot, focus groups were conducted with each of the three primary healthcare teams (14 participants, including eight GPs), and one with ECPs (five participants) and nine individual patient interviews. Data were analysed using a modified framework approach.

**Results:**

The impact of ECP home visiting on GP workload and patient care was perceived as positive by patients, GPs and ECPs. Initial preconceptions of GPs and patients about the ECP role and expertise, and reservations about the appropriacy of ECPs for home visiting, were perceived to have been overcome by the expertise and interpersonal skills of ECPs. Fostering a culture of collaboration between ECPs and GPs was instrumental to remodelling professional boundaries at the practice level.

**Conclusion:**

Broadening the skill-mix to incorporate extended-scope practitioners such as ECPs, to deliver primary care home visiting, presents an opportunity to increase resilience in the general practice workforce.

KEY MESSAGES

- In this pilot, patients, GPs and Emergency Care Practitioners (ECPs) felt that ECPs performing primary care home visits positively impacted patient care and GP workload.
- Overcoming preconceptions about ECP role and expertise, and remodelling professional boundaries between ECPs and GPs were particularly important.

## Introduction

Primary care services in the UK and Europe are facing a workforce crisis. In the UK, growth in the volume and complexity of general practice work has been compounded by falling general practitioner (GP) numbers [1,2]. Broadening the primary care workforce to reduce demand on GPs is one of 10 National Health Service England and Improvement (NHSE and NHSI) high-impact targets [3]. The British Medical Association and NHS England five-year plan (2019) proposed over 20,000 posts for allied health professionals [3], expanding their scope of practice to support primary care teams [2]. A community paramedic working in this extended scope role is a clinician with paramedic training with ‘community-focused extension of the traditional emergency response and transportation paramedic model [4]’, including primary care roles.

The delivery of medical care to homebound patients is integral to primary care in Europe. In the UK, GPs conduct planned and unscheduled (acute) home visits. These place a significant demand on GP time. Ambulance service clinicians, such as paramedics, are accustomed to assessing patients with acute health problems in their own homes, and may be ideally placed to take on primary care home visiting [5]. Potential benefits could include shorter waits for home visits, fewer emergency transfers to hospital, longer consultation times and increased patient satisfaction [6–8]. GPs, free from home visits, could have more time to manage other patient groups with complex needs [5,6].

There is policy support for expanding practitioners from other healthcare disciplines like paramedics into roles traditionally ascribed to GPs, despite limited evidence about the potential benefits or unintended consequences such as the impact on continuity of care [5,6]. A small qualitative study on paramedic practitioners performing home visits for patients aged >65 years found that patients deemed home visits by paramedics as acceptable, but poorly understood the role of paramedic practitioners [9].

In recent years, several professional groups have taken on new roles in UK primary care. The integration of nurse practitioners into primary care teams has been described as a ‘dynamic, complex and messy’ process [10], necessitating careful planning, collaboration and redefinition of professional boundaries [10–12]. More recent work on integrating physician associates in primary care suggests that professional boundaries can be redefined at the GP practice level [13]. Despite recent work exploring how paramedics may be deployed in primary care, there is a paucity of evidence about how to embed such practitioners into primary care teams [5,14].

Emergency care practitioners (ECPs) are practitioners from paramedic or nursing backgrounds, with extended scope and training to work across traditional organisational boundaries [15], such as in primary care roles. This study took the opportunity offered by a pilot of primary care home visits conducted by ECPs, to retrospectively explore patient and staff views and experiences of this model of home visiting, and answer the following research questions:

1. What are patient and primary care and ambulance staff perceptions of a home visiting service performed by ECPs in place of the GP?
2. What were the positive impacts and any unintended consequences?
3. What factors influenced collaborative working between GPs and ECPs?

## Methods

### Setting

Three general practices in a semi-rural area of northern England collaborated with the NHS Ambulance Trust to pilot primary care home visits conducted by ambulance service clinicians (role described below). The combined patient population across the three practices (January 2019) was approximately 25,500 (adjusted list sizes of 8293, 10,663 and 6650 patients). The rurality index and indices of deprivation scores of the three practices ranged between 1.019 and 1.074 and 9–10, respectively.

### ECPs

Each day there were two or three ECPs, employed by the local ambulance service, working with three GP surgeries to conduct home visits. They were drawn from a pool of 31 ambulance service staff. Half of the ECPs (15/31) had a UK paramedic background and half were nurses (16/31). The majority (24/31) had completed advanced clinical practice (MSc or equivalent) training, equipping them with common skills to expand their scope of practice [16] (for example primary care home visiting), and the remaining staff (7/31) were undertaking this training. Regardless of their background, all ECPs identified themselves as working for the ambulance service, wore the same uniform and performed the same role.

The term ECPs will be used throughout this paper to refer to this ambulance service staff group conducting home visits. The role performed matches that of an ECP; ‘a generic practitioner drawn mainly from paramedic and nursing backgrounds’ with ‘formal training and extended clinical skills’, to fulfil roles across traditional organisational boundaries and ‘to carry out initial assessment of patient need, and to either treat or refer to the appropriate care pathways’ [15].

### Pilot model of home visiting

This was a five-month pilot service (November 2018–March 2019), operating during routine GP working hours (Monday-Friday, 08:00-18:30). ECPs conducted home visits for patients requesting same-day home visits. Patients or carers telephoned the practices and provided brief details to reception staff about the reason for the home visit request. GPs ensured that there was no specific reason why allocation should not be to an ECP, for example, medical emergencies requiring immediate conveyance to hospital. The ambulance trust stipulated that the following patient groups were not eligible for an ECP visit due to the scope of their training; children under 5 years, pregnant women, mental health crises and patients with palliative care needs.

Over the five-month pilot, ECPs performed 857 home visits (440 Practice 1, 281 Practice 2 and 136 Practice 3), for patients between 21 and over 100 years old. The most common problem assessed was respiratory illness, followed by musculoskeletal symptoms (non-trauma), musculoskeletal/soft tissue injury and urinary symptoms. This is typical for same-day home visit requests [17].

### Sampling and recruitment

Semi-structured interviews were carried out with patients who received a home visit from an ECP. Eligible patients were identified by practice managers, in conjunction with GPs, at each surgery *via* clinical record screening. Practice staff worked in reverse chronological order, according to visit date, to maximise participant recall. Care home residents and patients unable to consent were ineligible for participation. Eligible patients were telephoned by non-clinical surgery staff to assess interest in the study. Participant information sheets and consent forms were posted, and followed up by a telephone call from the research team.

Focus groups were conducted with GP and ambulance service staff involved with the pilot. Practice managers identified potential primary care staff participants, and ECPs were identified by ambulance service management.

### Data collection

Data collection took place between May–October 2019 (Table 1). Topic guides were based on published literature and discussions with senior staff involved in the pilot. One-on-one interviews were employed for patients as their experience may involve sensitive discussions. Interviews with patients were conducted by RS/SR (experienced post-doctoral female researchers), by telephone or in the patient’s home. Informed written consent was obtained before interviews started. Competing priorities for one practice limited the recruitment of patients for interview. The interview topic guide was designed to elicit open-ended responses, with probes to encourage greater reflection in specific areas: how the ECP home visit compared to a GP home visit, views on continuity of care, patient experience of treatment decision-making. Interviews lasted 15–60 min. Data sufficiency was determined when no new subthemes emerged.

**Table 1.** Participant characteristics.

Interview type	Total participants
*Patients*	
GP Surgery 1	5
GP Surgery 2	3
GP Surgery 3	1
Total	9
Patient age range: 40–87 (7 female 2 male)	
*Staff*	
GP Surgery 1: Focus group GP (*n* = 4) Reception staff (*n* = 2) Nurse (*n* = 1)	7
GP surgery 2: Focus group GP (*n* = 3) Practice manager (*n* = 1) Reception staff (*n* = 1)	5
GP Surgery 3: Dyadic interview GP (*n* = 1) Reception team leader (*n* = 1)	2
ECPs: Focus group	5
Total	19
Grand total	28

Focus groups were utilised for staff participants, as they were known to each other, allowing participants to exchange views. Separate focus groups were conducted per physical workplace. The focus group topic guide explored expectations of ECP home visiting; whether expectations were met; communication between GPs and ECPs; perceived impact on patient care and primary care workload; and whether the pilot produced any unanticipated consequences.

Interviews and focus groups were audio-recorded and transcribed verbatim. Data collection ceased once data sufficiency was achieved; determined as when no new subthemes emerged.

### Data analysis

Transcripts were analysed using a framework approach [18], which provides a flexible and rigorous approach to qualitative analysis. A matrix was constructed in Microsoft Excel, with interview and focus group topics inserted as initial themes within the framework (as columns) and populated with data by the interviewers to ensure consistency and enhance reliability. RS and SR coded interview and focus group transcripts separately, then together, iteratively revisited the framework and its themes to review and re-shape themes as they emerged from coding. Themes were then populated within the framework.

## Results

Two separate focus groups were conducted with staff from two practices (one focus group per practice), plus a dyadic interview with a third practice, and one focus group with ECPs. Individual interviews were carried out with nine patients (Table 1).

**Figure 1.**
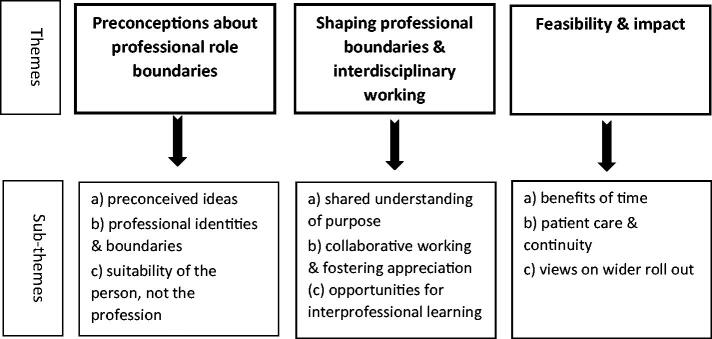
Themes and sub-themes.

Our analysis is summarised in Figure 1, according to the following themes and sub-themes.

### Theme 1: Preconceptions about professional role boundaries

This theme describes participant’s preconceptions about the ECP role and expertise, prior to the pilot commencing.

#### Preconceived ideas

While most GPs embraced the concept, some voiced reservations about the acceptability to patients of ECP home visiting. They believed that patients may have certain expectations about who visited, and prefer continuity with GPs.

*‘…we were a little bit concerned about patient acceptance of them… It’s quite traditional around here isn’t it? Our general practice, they’re (patients) used to seeing familiar faces’. Participant 1, GP, Practice 3.*

Practice staff also expressed concerns that the ECP clinical expertise may be narrower than GPs. At the outset, the skillset, previous experience and training of ECPs were not fully appreciated.

*‘I was a little bit concerned about the level of knowledge and expertise …often our patients have lots of comorbidity and they are on a lot of drugs so actually probably the most complicated group of people that we see…’. Participant 3, GP, Practice 2.*

*‘… initially when it was all proposed it was like, “‘Well who are these people? What are their skill levels? Which patients are we going to give them?”’ Participant 5, GP, Practice 2.*

These perceptions were revised as the pilot progressed.

*‘…I suppose we did have a degree of apprehension that they might be a little bit risk averse when dealing with patients because as you say, they’re not really used to making that many management decisions – just more stay at home or sending to hospital. But we certainly didn’t find that [when the pilot started]. For the most part they were keeping people at home appropriately with appropriate safety net advice… I think that the care they provided was excellent.’ Participant 1, GP, Practice 3.*

Patients also held preconceived ideas about the role of ambulance service staff, and that the arrival of an ECP meant they were sufficiently unwell to require hospitalisation. Reception staff aimed to explain to patients that the home visit may be performed by an ECP. However, many patients reported being unaware that an ECP would arrive. Patients also worried that ambulance service staff were being taken away from their emergency response role.

*‘…she (receptionist) said, ‘we’ll send someone out,’ and I assumed it was a doctor but obviously it wasn’t, so I was very surprised […] I thought, ‘Is there something wrong with me, worse than what I actually said over the phone?’ Ivy, patient, Practice 1 (names used are pseudonyms).*

*‘…I was thinking, them coming to me, they were called away, then someone who was experiencing a life-threatening thing – were they able to get to them…?’ Lisa, patient, Practice 1.*

#### Professional identities and boundaries

The professional identity of GPs and ECPs is a key sub-theme. When discussing skill sets, both GPs and ECPs engaged in boundary work, defining the other as outside their profession’s boundary. GPs positioned themselves as particularly capable in dealing with medical complexity, fearing that ECPs would be more inclined to admit patients to hospital.

*‘I think, as a GP, one of our skills is risk management and managing complexity and how we really didn’t know what the quality of the practitioners would be, and how they would manage with that with our population in that regard… would [hospital] admissions go up?’ Participant 3, GP, Practice 1.*

In turn, ECPs highlighted situations where their knowledge of urgent care pathways placed them in a better position to organise efficient transfer to hospital.

*‘Depending on how they need to go in. It could be a transport only into hospital and are appropriate for a certain type of vehicle, which the GP might not know about’. Participant 2, ECP Focus Group.*

Initial fears and preconceptions of GPs about the capabilities of the ECPs were resolved throughout the pilot, as described below.

Primary care staff and patients referred to ECPs as ‘paramedics’ or identified them according to the organisation they worked for. Despite some practitioners having a nursing background, as opposed to paramedic training, ECPs performed the same role, so distinctions were not made between paramedics and nurses. There was limited awareness of primary care staff of the differing professional backgrounds:

*‘they were advanced practitioners, and [I discovered after the pilot started that they] might have a nursing background or an entirely separate background from the sort of ambulance paramedic stream so that’s a preconception again’. Participant 3, GP, Practice 1.*

#### Suitability of the person, not the profession

Patients had positive views of ECP’s expertise and interpersonal skills. Patients felt the treatment received from the ECPs was thorough and comparable with that provided by a GP.

*‘You’re looking for a qualified person you know… To have a qualified person coming in just gives you that wee bit of confidence, so it could be a paramedic, it could be a doctor, it could be a nurse’. Jane, patient, Practice 1.*

Interpersonal skills and relationships were key for patients:

*‘…he instantly put my husband and I at ease. He was very friendly and he had a nice smiley face. He didn’t come in with – I’m gonna say a typical doctor’s look on his face [chuckles] ’. Ethel, patient, Practice 2.*

A minority of patients preferred a GP home visit, highlighting continuity and the assurance of familiarity. One patient with a complex medical background, requiring regular home visits, reflected on the value of continuity:

*‘I feel very vulnerable, lying in bed as well, letting people in to your private… But she [ECP] made me feel comfortable […] I would prefer somebody who I know and who knows my history, to be honest. […] I wouldn’t mind a paramedic, but a doctor is best. And with my circumstances, it’s probably a better idea [to have a doctor]’. Amy, patient, Practice 3.*

### Theme 2: Shaping professional boundaries and interdisciplinary working

This theme describes how, at the individual practice level, the potential for preconceptions and professional boundaries to impede interdisciplinary working were navigated.

#### Shared understanding of purpose

Practice staff and ECPs held positive views of the pilot. The expected benefits of enhancing skill-mix, such as reducing GP workload and learning opportunities, were highlighted.

*‘Initially obviously we were really positive about it because […] we get a lot of home visits and anything that could be done to try and reduce that workload’. Participant 1, GP, Practice 3.*

*‘…[what] I was sold on was that we were going to be doing more primary care work, working alongside GP surgeries… I don’t want to be going around on the blue lights’. Participant 4, ECP Focus Group.*

The three GP practices had the opportunity to shape the way ECPs integrated into their teams.

*‘It meant that the scale – we could be involved a little bit in the design of how information flowed and how we communicated’. Participant 1, GP, Practice 1.*

#### Collaborative working and fostering appreciation

Working collaboratively was viewed as instrumental to the success of the pilot. Both ECPs and GPs reflected on the importance of positive communication, as well as trust and appreciation of each other’s practices, which developed over the course of the pilot.

*‘[…] initially when we first went in there was – what they were giving us was sort of […] the quite simple ones […] we were capable of doing more…’. Participant 5, ECP Focus Group.*

*Participant 4: ‘I think trying to get to that level of trust’…*

*Participant 5: ‘yeah […] by the end of the pilot it was just go and see almost anything!’ Participant 5, ECP Focus Group.*

*‘It was apparent – the quality of assessments people were getting [from the ECPs] was really high– and the judgements, it all sounded appropriate’. Participant 3, GP, Practice 1.*

Patients were also reassured by collaboration between GPs and ECPs, which maintained a level of continuity of care.

*Researcher: ‘How did you feel about the paramedic (ECP) calling your GP just to check?’*

*Ellen: ‘Well it was reassuring that you’re getting the right medication, because obviously your doctors know, you know? They’ve got all your information in front of them’. Ellen, patient, Practice 2.*

#### Opportunities for interdisciplinary learning

The pilot created an opportunity for mutual teaching and learning. However, much of this learning was one way, with ECPs typically taking on the student’s role while GPs viewed themselves as teachers. Debriefs allowed ECPs to learn about primary care, affirm their decision making and fostered working relationships.

*‘It became very much an educational role that you had with [the ECPs]… They were learning on the job and you were kind of educating them….’ Participant 5, GP, Practice 2.*

### Theme 3: Feasibility and impact

This theme describes views on the impact of ECPs performing home visits in place of GPs.

#### The benefits of time

GPs considered their home visiting responsibilities to be time-consuming. There was a perception that ECPs released time for GPs to do other tasks.

*‘I think [the pilot] reduced the amount of acute visits that we [GPs] had to do… So I still visited quite a lot during the period but rather than seeing acute visits, I had more opportunity to do some of the management stuff or care planning’. Participant 5, GP, Practice 2.*

Patients and GPs also appreciated the timeliness of ECP home visits, and the amount of time spent with patients – whether this was just to be listened to, or because the ECPs had time to provide a more thorough assessment.

*‘[…] Basically, we had the time to do that. GPs don’t have the time to do that and I think that more than compensated for the fact that the patient was getting a practitioner (ECP) instead of the GP’. Participant 4, ECP Focus Group.*

*‘[…] so going out to see them it’s not just about giving them the antibiotics and steroids if that’s what they need and leaving them at home […] We’ll see how they use their inhalers’. Participant 1, ECP Focus Group.*

#### Patient care and continuity

Despite the perceived benefits to patient care, there were some home visits that GPs felt would be more appropriate for them to conduct. They felt that the ECPs’ skills, rooted in acute care, placed limits on their decision-making.

*‘I think one of the shortcomings would be some of the things that were outside of [the ECP’s] knowledge or skills set […] musculoskeletal things, somebody’s got a sore knee, they would give them analgesics and so forth and maybe some physio. They’re not going to be thinking about further referral, knee imaging, steroid injections so I think that would then come to us. Appropriately’. Participant 3, GP, Practice 1.*

Care home staff were described as broadly receptive to ECPs conducting home visits but there were instances where staff specifically requested a GP, for more complex or ongoing issues or for residents near to end of life.

*‘Occasionally one or two of the care homes might have said ‘Not the paramedic,’ and that wasn’t personal…I think they just wanted a GP just to cast an eye for peace of mind as much as anything’. Participant 1, GP, Practice 2.*

##### Views on wider roll-out

All interviewees supported a wider roll out of the ECP home visiting pilot. Some suggested minor amendments to how ECPs were embedded into practices, to maximise efficiency.

*‘I think that it would be much better if [ECPs] were integrated in our own teams rather than coming from outside. […] Just because I think we would be able to design a better service than they (service commissioners) could do, include them to do other things like the acute surgeries, in house training, getting the feel of how we work as a team’. Participant 1, GP, Practice 1.*

## Discussion

### Main findings

This article presents novel qualitative data on the experiences of healthcare staff and patients of home visits performed by ambulance service ECPs, with a paramedic or nursing background. Primary care and ambulance service staff felt that ECPs successfully integrated into primary healthcare teams and worked collaboratively with GPs, relieving some GP workload pressure. The impact on patient care was perceived to be positive by primary care and ambulance service clinicians as well as patients, although there were examples where continuity of care by GPs may be preferred by patients, especially if they felt they had complex health needs. Initial preconceptions of primary care staff and patients about the professional identity, and reservations about the appropriacy of ECPs to the home visiting role were overcome by the expertise and interpersonal skills of ECPs. Fostering a culture of collaboration between ECPs and GPs was felt by these staff groups to be instrumental in remodelling professional boundaries between GPs and ECPs.

### Links with the existing evidence base

This study provides important insights into the integration of alternative, extended-scope practitioners to GPs in the delivery of medical care to homebound patients, an important consideration for primary care in the UK and European countries [19]. Our work adds to the emerging evidence of practitioners, such as paramedics and ECPs, performing primary care healthcare roles, which describes positive experiences of health professionals and patients [5,8,9,14].

The importance of role perception and professional boundaries, and the factors that contributed to successful interdisciplinary working between GPs and ECPs, was similar to previously described for practitioners from non-medical professional groups extending their scope into primary care. Initially, GPs had reservations about transferring the responsibility for home visits to ECPs. The reservations stemmed from preconceptions about the ECP skill set and the existence of traditional professional identities, which have also been described when nurses have adopted roles traditionally ascribed to doctors [12]. The initial uncertainty described by GPs was mitigated by practices having the opportunity to define the scope of the ECP’s work, such as triage decisions and interdisciplinary learning, which is also an important feature of integrating nurse practitioners into primary care teams [10]. Collaboration between GPs and ECPs was another essential factor that mitigated the potential for professional role boundary conflicts [10,11,20]. As previously described for interdisciplinary working between physician associates and GPs [13], ECPs and GPs worked together to define their roles and reframe their professional boundaries at the GP practice level.

### Implications

This study provides important insights into the integration of paramedics and nurses into primary care teams to perform roles traditionally ascribed to GPs, such as home visiting. First, advanced planning is important to overcome common preconceptions about the expertise and role [10] of different professional groups. Second, as described previously [9], it is important to educate patients about the roles for alternative clinicians to GPs, such as ECPs, who are working in primary care roles. Third, the unique setting and array of challenges facing different practices – rurality, different practice sizes and workforce composition [6] – means that it is important for primary care teams to have the opportunity to shape interdisciplinary working with colleagues from other healthcare disciplines.

### Strengths and limitations

Our study’s strength was that it captured the experiences of primary healthcare and ambulance service ECP staff, as well as patients. However, these experiences relate to three neighbouring practices in northern England so the applicability of findings to different primary care settings may vary. One GP practice found patient recruitment challenging, which may have impacted on how their experience is represented in our analysis. Originally, the ECPs had all trained as either a UK paramedic or nurse. Our study participants were not familiar with the ECP’s background and we did not set out to detect differences in the experiences of healthcare professionals and patients between these two groups. Reservations expressed by care home staff about ECPs performing care home visits were reported by practice staff. Our study did not directly capture the experiences of care home staff or residents (due to the need for a more extensive research ethics process that was unfeasible in the study period). This represents an important area for future research.

### Future work

Further research is required to assess the impact on health and healthcare outcomes, such as conveyance to hospital, of practitioners from alternative healthcare disciplines (e.g. paramedics and nurses) performing home visits in place of GPs, and to explore the potential of such practitioners to take on other primary care roles, such as consultations in GP practices.

## Conclusion

Broadening the skill-mix to deliver primary healthcare to homebound patients represents an opportunity to increase resilience in the general practice workforce. It is essential to address preconceptions of patients and professionals about the role and expertise of paramedics and nurses, as they adopt roles traditionally ascribed to GPs. Remodelling professional boundaries with GPs as practitioners from other disciplines take on extended scope roles, is key.

## Supplementary Material

Supplemental Appendix 2: Staff focus group topic guide

Supplemental Appendix 1: Patient interview topic guide
