# Supplementary material for: Future-proofing the primary care workforce: A qualitative study of home visits by emergency care practitioners in the UK
Source: Eur J Gen Pract. 2021 May 12;27(1):68–76. doi: 10.1080/13814788.2021.1909565 (PMC8118426; doi:10.1080/13814788.2021.1909565)
Supplement: Supplemental Appendix 2: Staff focus group topic guide [file IGEN_A_1909565_SM8613.docx]

**Appendix 2: Staff focus group topic guide**

1. **So, the ambulance service practitioner intervention aside, how do you feel about home visits?**

**Prompt:** What is patient demand for home visits like at this practice?

Are there patients that request home visits more than others? What are these patients like?

How can home visits impact on your working week?

1. **What did you think when you first heard about ambulance service practitioners (ECPs) carrying out home visits?**

**Prompt:** What were your expectations?

*Could you expand on X?*

1. **What was it like when the pilot first started?**

**Prompts:** Were your expectations met?

Were there any teething problems?

Anything unexpected happen?

Did you feel sufficiently prepared for the pilot?

*Could you expand on X?*

1. **How about once the pilot had been ongoing for a while?**

**Prompts:** Did time iron out any teething problems?

Were there improvements?

Did X issue get worse?

*Could you expand on X?*

What about patient demand for home visits? Did this change with the introduction of the trial?

1. **How did you find the communication between the practice and ambulance service practitioners (ECPs)?**

**Prompt:** Turning to the GPs here, how much did you support the ambulance service practitioners (ECPs) in decision making?

How confident did you feel in the clinical assessments made by the ambulance service practitioners (ECPs)?

How might this have been improved?

Could you expand on X? / Why do you think that is?

1. **How about patient care? Did the pilot have any impact on this?**

**Prompt:** Could you expand on X? / Why do you think that is?

What about continuity of care**?**

1. **How were requests for home visits triaged to an ambulance service practitioner (ECP)?**

**Prompts:** How did you decide which patients were suitable for a NEAS visit, and which you felt you should visit yourselves?

From a reception perspective, what was it like?

Could that have been improved?

Could you expand on X? / Why do you think that is?

1. **Have any patients said anything to any of you about their experience of receiving a home visit from ambulance service practitioners (ECPs)?**

**Prompt:** Have any patients said anything?

Could you expand on X? / Why do you think that is?

1. **How did the pilot impact on your working day?**

**Prompt:** And general workload in the practice?

Consider before, during, after.

1. **If you could change anything about how the pilot worked, what would it be?**

**Prompts:** Could you expand on x?

Have you had any thoughts about a better way to manage acute visiting?

Could you expand on X? / Why do you think that is?

1. **Would you support the roll out of community paramedics (ECPs) in the same way across primary care?**

**Prompt:** Could you expand on X? / Why do you think that is?

*Sum up the last discussion*

*Ask the participants whether they have any questions or anything further to add.*

*Thank the participants for their time.*

**End of focus group.**
