# Supplementary material for: Future-proofing the primary care workforce: A qualitative study of home visits by emergency care practitioners in the UK
Source: Eur J Gen Pract. 2021 May 12;27(1):68–76. doi: 10.1080/13814788.2021.1909565 (PMC8118426; doi:10.1080/13814788.2021.1909565)
Supplement: Supplemental Appendix 1: Patient interview topic guide [file IGEN_A_1909565_SM8543.docx]

**Appendix 1: Patient interview topic guide**

1. **How do you feel about the home visits you get from [GP surgery] in general?**
   1. How often do you receive home visits?
   2. Do you usually see the same GP?
2. **Thinking back to earlier this year, when the GP receptionist told you that you may receive your home visit** **from an ambulance service practitioner (ECP), what was your initial reaction?**
   1. What were your expectations?
   2. Did you have any concerns?
3. **When the ambulance service practitioner (ECP) arrived at your house, what happened?**
   1. How quickly did they get there?
   2. What did they explain to you about why they had come and not a GP?
   3. Did they come in a marked vehicle? What did you think about that?
4. **What did the ambulance service practitioner (ECP) do in terms of treatment?**
   1. Did you need to go to hospital?
   2. Were you given any medication?
      1. Did they have what you needed, with them?
   3. Were they aware of your medical history?
5. **How did the visit compare to GP visits you’ve had in the past?**
   1. How did you feel about the ambulance practitioner visiting you, instead of your usual GP?
      1. Why/why not?
   2. How confident did you feel the decisions that were made about your care, by the ambulance practitioner (ECP)?
6. **Was the problem that you called about addressed?**
   1. If you had the same problem again, would you have a preference as to whether the visit came from a GP or an ambulance service practitioner (ECP)?
7. **Was there any follow up from the visit?**
   1. Were you telephoned by your GP after the visit?
   2. Were you referred to any other services, or the hospital?
   3. How do you feel about that?
8. **What are your overall views of the visit you received?**
   1. If the pilot was rolled out as a permanent way of doing things, would you be supportive? Why/why not?
   2. What would you change about your visit, if anything?
